# Influence of Oleic Acid on Rumen Fermentation and Fatty Acid Formation *In Vitro*

**DOI:** 10.1371/journal.pone.0156835

**Published:** 2016-06-14

**Authors:** Duanqin Wu, Liwei Xu, Shaoxun Tang, Leluo Guan, Zhixiong He, Yongjuan Guan, Zhiliang Tan, Xuefeng Han, Chuanshe Zhou, Jinhe Kang, Min Wang

**Affiliations:** 1 Institute of Subtropical Agriculture, The Chinese Academy of Sciences, Changsha, Hunan 410125, P.R. China; 2 Institute of bast fiber crops, Chinese Academy of Agricultrial Sciences, Changsha, Hunan 410205, P.R. China; 3 Department of Agricultural, Food and Nutritional Science, University of Alberta, Edmonton, Alberta, Canada; 4 UWA Institute of Agriculture M082, University of Western Australia, 35 Stirling Highway, Crawley WA 6009, Australia; Agricultural University of Athens, GREECE

## Abstract

A series of batch cultures were conducted to investigate the effects of oleic acid (OA) on *in vitro* ruminal dry matter degradability (IVDMD), gas production, methane (CH_4_) and hydrogen (H_2_) production, and proportion of fatty acids. Rumen fluid was collected from fistulated goats, diluted with incubation buffer, and then incubated with 500 mg *Leymus chinensis* meal supplemented with different amounts of OA (0, 20, 40, and 60 mg for the CON, OA20, OA40 and OA60 groups, respectively). Incubation was carried out anaerobically at 39°C for 48 h, and the samples were taken at 12, 24 and 48 h and subjected to laboratory analysis. Supplementation of OA decreased IVDMD, the cumulative gas production, theoretical maximum of gas production and CH_4_ production, but increased H_2_ production. However, no effect was observed on any parameters of rumen fermentation (pH, ammonia, production of acetate, propionate and butyrate and total volatile fatty acid production). The concentrations of some beneficial fatty acids, such as cis monounsaturated fatty acids and conjugated linoleic acid (CLA) were higher (*P* < 0.05) from OA groups than those from the control group at 12 h incubation. In summary, these results suggest that the OA supplementation in diet can reduce methane production and increase the amount of some beneficial fatty acids *in vitro*.

## Introduction

Ruminants are capable of using coarse fodder through rumen microbial fermentation and providing high quality products (meat and milk) for humans. However, rumen fermentation is associated with the formation of methane (CH_4_), which is one of effective greenhouse gases, and further results in a part loss of energy of diet [1]. Dietary manipulation, such as adding “proper” additives in feeds, has been considered a feasible way to decrease the enteric CH_4_ emissions of ruminant. The long-chain fatty acids, especially unsaturated fatty acid (UFA), have toxic effects on rumen methanogenic archaea which lead to the reduction of CH_4_ production [2–3]. Previously, Beauchemin *et al*.[4] reported that several lipid sources (i.e., tallow, sunflower oil, and whole sunflower seeds) which contained mainly long-chain fatty acids (LCFA) reduced CH_4_ emissions by an average of 17% from growing cattle. Similarly, dietary supplementation with unsaturated fatty acid (UFA) has been reported to decrease CH_4_ production *in vitro* [5–6].

In ruminants, dietary fatty acids are extensively hydrogenated and isomerized by rumen microbes, which can contribute to the lipid profile of ruminant products [7–8]. Oleic acid, also typical in ruminant diets, is one of the fatty acids for animal and can be hydrogenated by microbes in the rumen. It has been indicated that oleic acid (OA) could be biohydrogenated to C18:0 when incubated with mixed or pure cultured rumen bacteria [9]. Contrastively, some studies indicated that rumen bacteria isolated from sheep could isomerize OA and produce trans-10-C18:1 or trans-11-C18:1 using *in vitro* system [10–11]. Isomerization and conversion of OA to other C18:1 intermediates were highly variable in the rumen due to the bacterial diversity [12]. To our knowledge, little information about effects of OA on rumen fermentation and production of fatty acid isomers is available during the process of *in vitro* incubation with mixed rumen microbes from the goats. We hypothesize that OA supplementation in the substrate of *Leymus Chinensis* could produce associative beneficial effects for CH_4_ emission and fatty acid isomers production *in vitro*. Therefore, under the controlled conditions of an *in vitro* experiment, the objective of this study was to analyze the variation of dietary OA supplementation in *in vitro* CH_4_ production, fermentation parameters and fatty acid isomers production.

## Materials and Methods

This study was carried out in strict accordance with the recommendations in the Guide for the Care and Use of Laboratory Animals of the National Institutes of Health. The protocol was approved by the Committee on the Ethics of Animal Experiments of the Institute of Subtropical Agriculture, the Chinese Academy of Sciences, Changsha, China. (Permit Number: 2013–10). All surgery was performed under sodium pentobarbital anesthesia, and all efforts were made to minimize suffering.

### Animals and Rumen Fluids Collection

Four adult Liuyang black goats (a local breed in southern China) fitted with permanent rumen cannulas were used in the study. The goats, with the body weight of 25 ± 2.0 kg, were penned individually in stainless metabolic cages, and had free access to fresh water throughout the entire experiment. Diets containing approximately 70% roughage and 30% concentrate were formulated (DM basis) and offered twice in equal amounts at 08.00 and 20.00 h to supply 1.3 times maintenance requirement of metabolizable energy according to our previous studies [13]. Before the morning feeding, rumen fluid was collected from each goat using a manual vacuum and immediately transferred into pre-warmed (39°C) thermos flasks (2 L) that was filled with CO_2_. The rumen fluid of the four goats were then mixed and filtered through four layers of cheesecloth under continuous flushing with CO_2_.

### In Vitro Incubation

The *in vitro* incubation system was set up using the method described by Tang *et al*. [14]. The rumen fluid was mixed (1/4, v/v) with a buffer solution [15]. Substrate (*Leymus chinensis* meal mainly contains crude protein 8.20%, crude fat 2.17%, crude ash 4.58%, neutral detergent fibre 66.57% and acid detergent fiber 38.75%, 0.50 g) and 50 mL of the rumen fluid and buffer solution mixture were added to a glass gas-tight incubation flask under anaerobic conditions. Experiments were divided into four treatments: 0, 20, 40 and 60 mg OA/50 ml culture solutions, namely CON, OA20, OA40 and OA60 respectively. The OA in each treatments was emulsified with 1 mL of ethanol before incubation *in vitro*. The flasks were filled with CO_2_ and incubated in a shaking water bath at 39°C for 12, 24 or 48 h. Triplicate flasks were used for each sampling time point. At 12 h and 24 h of incubation, three flasks from each treatment were taken out and immediately frozen. In addition, three flasks containing only incubation medium were also incubated as blanks to correct gas production resulting from rumen fluid activity. The *in vitro* fermentation was separately run three times on different days of collecting mixed rumen fluids, so that each treatment was conducted in triplicate.

### Sampling and Measurements

*In vitro* gas production (GP) was recorded continuously by an automated system which connected with the fermentation bottle via electric multi-channel pressure transducer (CYG130-12, Kunshan M&C Technology Co., LTD, China).

The 5 mL of gas in the headspace were sampled from each bottle at 12, 24, and 48 h of incubation, and injected into the vacuum flask (Labco Exetainer, UK) for CH_4_ and hydrogen (H_2_) determination by gas chromatography (GC7890A, Agilent, USA) using the method described by Wu *et al*. [16], respectively.

The pH of incubation fluids was measured immediately after the removal of fermentation flasks using a pH meter (Model PHS-3C, Shanghai precision & scientific instrument Co., LTD, China). The *in vitro* dry matter digestibility was measured using the method described by Wu *et al* [17].

Samples for volatile fatty acid analysis (4 mL) were acidified with 1 mL 25% metaphosphoric acid solution and centrifuged for 15 min at 10,000 × *g* at 4°C. The supernatant was recovered, transferred to vials and analyzed by gas chromatography (GC7890A, Agilent, USA), according to Wu *et al* [16]. Ammonia-nitrogen (N) was determined using the method described by Weatherburn [18].

The fatty acids of incubation fluids were extracted using the method described by Bligh and Dyer [19] and stored at -20°C until use. Lipids were methylated after the addition of C13:0 as an internal standard according to the method of Ichihara *et al* [20]. subjected to a gas chromatography equipped with a flame ionization detector and a fused silica capillary column (100 m × 0.25 mm × 0.2 μm) coated with 100% cyanopropyl polysiloxane (CP-Sil 88, Chrompack; Middelburg, The Netherlands). The initial temperature of the oven was set at 45°C for 4 min, then increased to 175°C at a rate of 13°C/min and maintained for 27 min, further raised to 215°C at a rate of 4°C/min and finally kept constant at 215°C for 35 min. Analysis of all peaks was accomplished by comparing their retention time with FAME standards.

### Statistical Analysis

GP profiles were obtained after fitting the data to the logistic-exponential equations of Wang *et al*.[21]:

$$GP_{\text{t}}=vf\times \frac{1-\text{exp}(d-t\times k)}{1+\text{exp}(b-k\times t)}\;V_{0}=vf\times \frac{1-\text{exp}(d)}{1+\text{exp}(b)}$$

$$FRD_{0}=\frac{\text{k}}{1+\text{exp}(b)}\;t_{0.5}=\frac{\text{ln}(2+\frac{1}{d})}{k}$$

where GP_t_ represents gas production at *t* time point, and *vf* is the final asymptotic gas volume (mL); *k* represents fractional rate of gas production; *d* and *b* are constants; *t* represents incubation time (h); V_0_ represents the initial gas volume at *t* = 0; FRD_0_ represents initial fractional rate of degradation at *t* = 0; t_0.5_ represents the time at which half of the final gas production is generated.

The volume of gas CH_4_ (gCH_4_) or gas H_2_ (gH_2_) produced between two consecutive incubation times and accumulative gH_2_ produced could be calculated as the mathematical equations of Wang *et al*.[22]:

$$\Delta V_{H}\text{i}=\Delta V_{H\text{gi}}+\Delta V_{Hhi}=\rho P_{i}C_{Hi}+V_{h}\left(C_{H\text{i}}-C_{H(\text{i-1})}\right)$$

$$\Delta V_{H\text{n}}=V_{H(n-1)}+\Delta V_{Hn}=\;\sum_{i=1}^{i=n}\Delta V_{HI}=\sum_{i=1}^{i=n}\rho P_{i}C_{Hi}+V_{h}C_{Hn}$$

where ΔV_Hi_ is the volume (mL) of gCH_4_ (gH_2_) produced between t_i-1_ and t_i_; ΔV_Hgi_ is gCH_4_ (gH_2_) produced and presented in the gas released; ΔV_Hhi_ is the gCH4 (gH_2_) produced and presented in the head-space gas; P_i_ is the pressure (kPa) measured by the sensor at t_i_; ρ is the constant linking gas volume and pressure measured by the sensor and its value is slightly different for different sensors; C_Hi_ is the gCH_4_ (gH_2_) concentration in gas measured by the gas chromatograph at t_i_, and the initial gCH_4_ (gH_2_) concentration is zero with ‘C_H0_ = 0’; V_Hn_ (ml) is the accumulative gCH_4_ (gH_2_) produced between ‘t = 0’ and t_n_; i and n are the number of venting events, and i≤n; V_h_ is the volume (ml) of headspace in the flask.

Data were subjected to the MIXED procedure of SAS 9.2. Total gas production and gas production profiles were analyzed as a completely randomized design. The model included the fixed effect of treatment. For CH_4_, H_2_ production and long-chain fatty acid isomers, the statistical model included the fixed effects of treatment and treatment × incubation time. The statistical significance of the differences between means was tested using the Tukey’s test. Means were expressed using least square means and presented with the standard error of the means (SEM). Statistical significance was declared at *P* ≤ 0.05.

## Results

### In Vitro DM Disappearance

**Fig 1:**
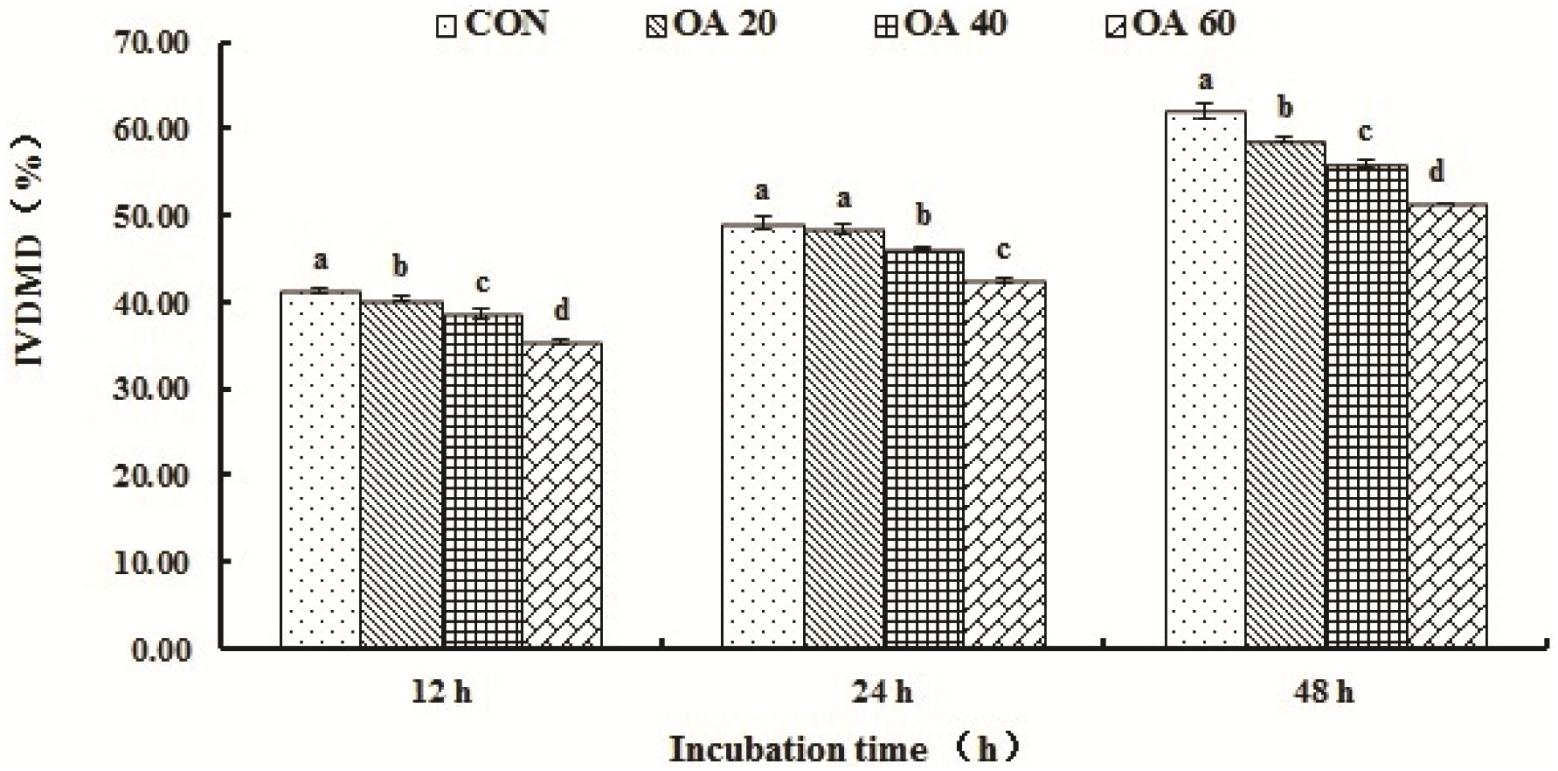
The *in vitro* dry matter degradability of *Leymus chinensis* in response to different levels of OA supplementation. All values are mean ± SEM. ^a, b, c, d^ Means that are sharing different superscripts are different (*P* < 0.05) within the same incubation stage.

The IVDMD of *Leymus chinensis* of CON, OA20, OA40 and OA60 groups are shown in Fig 1. The IVDMD decreased (*P* < 0.05) in response to OA supplementation at 12 h as well as at the end of incubation *in vitro*. While at 24 h, the IVDMD of CON and OA20 groups were not different (*P* > 0.05), but higher (*P* < 0.05) than those of OA40 and OA60 groups.

### In Vitro Gas Production and the Model Parameters

**Fig 2:**
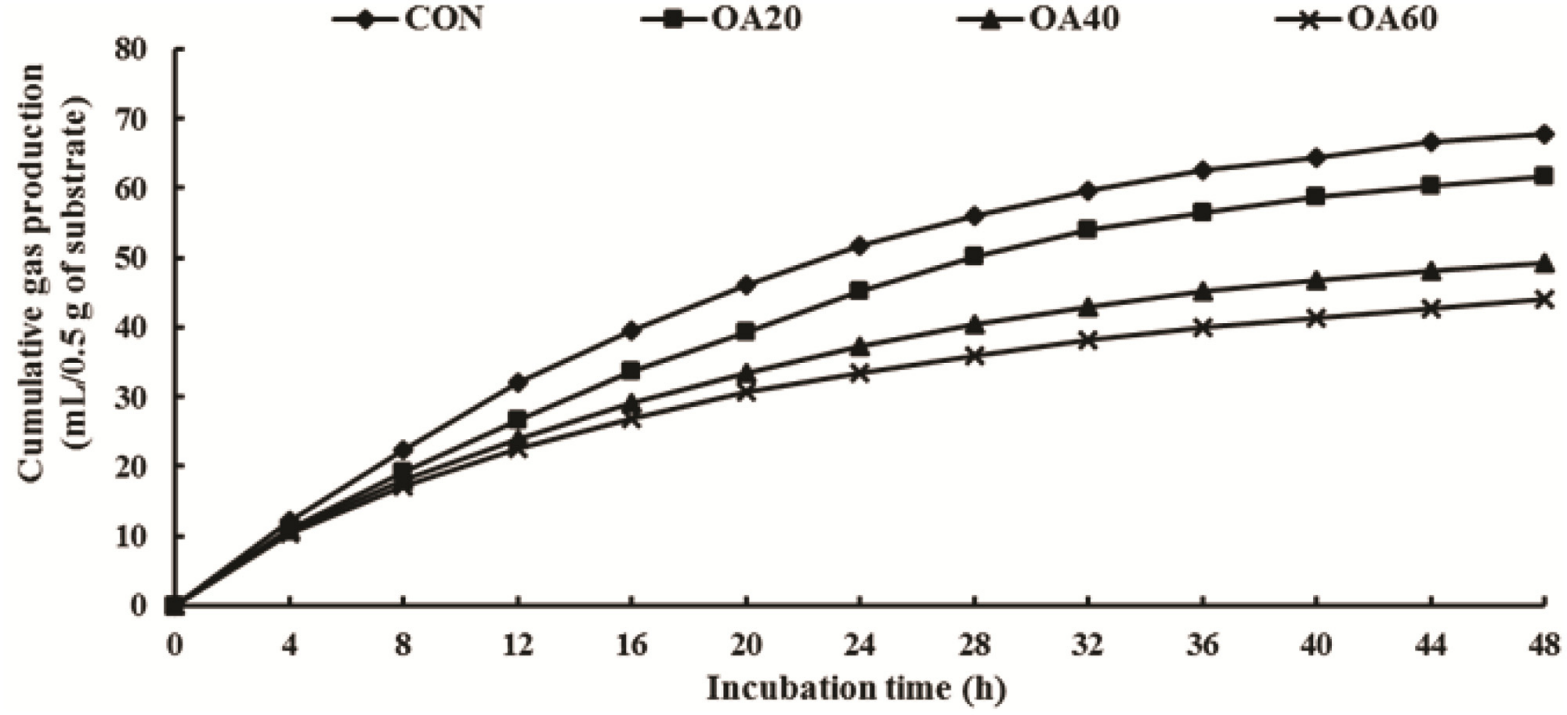
*In vitro* accumulative gas production of *Leymus chinensis* supplemented with OA.

GP and the model parameters are shown in Fig 2 and Table 1, respectively. From Fig 2, we found that GP was decreased (*P* < 0.05) in response to OA supplementation during the whole *in vitro* incubation period. From Table 1, we observed that the logistic-exponential equation fit well to the GP for all the treatments (R^2^ = 0.99). Decreased GP respond to OA supplemented in the substrate, and GP of CON in fermentation was higher (*P* < 0.05) than OA40 and OA60 group. From the value of FRD_0_, we noted that the initial fractional rate of degradation of *Leymus chinensis* in CON was higher (*P* < 0.05) than OA supplemented group. With the increase of OA dose, t_0.5_ value also gradually increased, but the substrate degradation of *Leymus chinensis* gradually slowed down.

**Table 1:** Effects of OA supplementation on the gas production parameters *in vitro*.

	CON	OA20	OA40	OA60	SEM
*vf*	66.39^a^	64.28^a^	51.34^b^	47.93^b^	1.22
b	0.72^b^	1.35^a^	1.29^a^	0.52^c^	0.05
FRD_0_ (×10^−2^)	3.23^a^	2.07^c^	2.07^c^	2.63^b^	0.07
t_0.5_	14.13^c^	17.73^b^	18.08^a^^b^	18.42^a^	0.15
R^2^	0.99	0.99	0.99	0.99	

^a, b, c^ Within a row, means with different superscripts are different (*P* < 0.05).

SEM = standard error of the means. *vf* means final asymptotic gas volume (mL); b is constant; FRD_0_ represents initial fractional rate of degradation at t = 0; t_0.5_ represents the time at which half of the final gas production is generated.

### In Vitro CH_4_ and H_2_ Production

**Fig 3:**
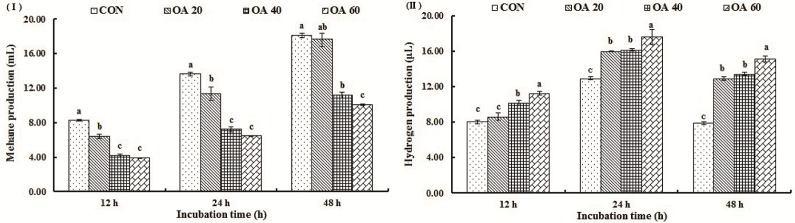
Effects of OA supplementation on CH_4_ (I) and H_2_ production (II) of *in vitro* incubation. All values are mean ± SEM. ^a, b, c^ Means that are sharing different superscripts are different (*P* < 0.05) within the same incubation stage.

With the increase of incubation time, cumulative production of CH_4_ of all the treatments was increased as well. Cumulative CH_4_ production was affected (*P* < 0.05) by treatments during the whole incubation period, except between CON and OA20 at the end of the incubation (Fig 3I). H_2_ production *in vitro* increased from 12 h to 24 h, but notably declined at 48 h, and was the highest (*P* < 0.05) in OA60 group through the whole incubation period (Fig 3II).

### In Vitro Fermentation parameters

**Fig 4:**
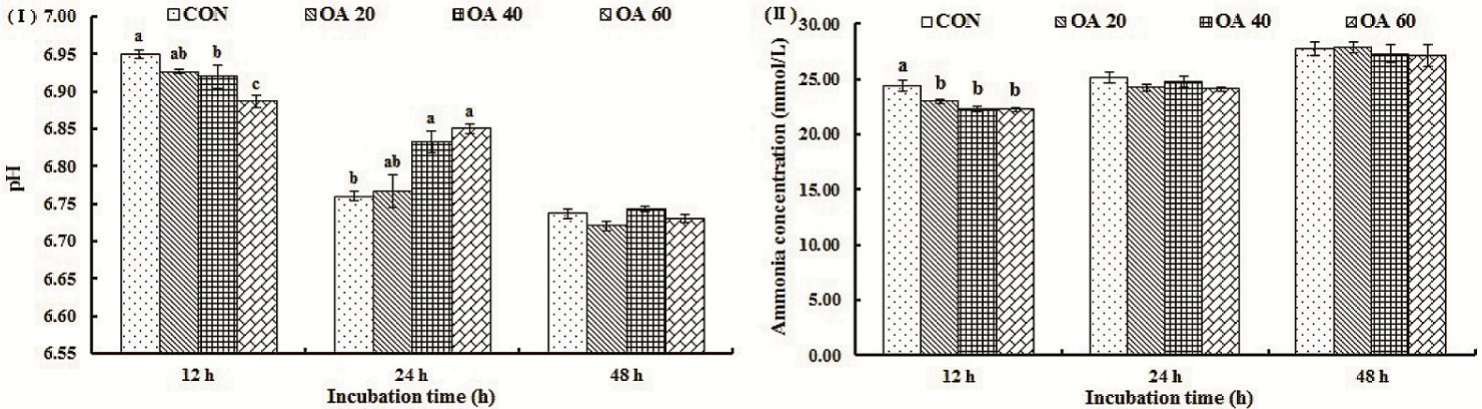
Effects of OA supplementation on pH (I) and NH_3_-N concentration (II) of fermentation liquor *in vitro*. All values are mean ± SEM. ^a, b, c^ Means that are sharing different superscripts are different (*P* < 0.05) within the same incubation stage.

For all treatments, pH gradually reduced with the increase of incubation time (Fig 4I). Ruminal pH of OA40 and OA60 group was higher (*P* < 0.05) than CON at 24 h time point, but was not different (*P* > 0.05) by the treatments. NH_3_-N concentration at 12 h was higher (*P* < 0.05) in CON group, but not different (*P* > 0.05) at 24 and 48 h time point (Fig 4II).

**Fig 5:**
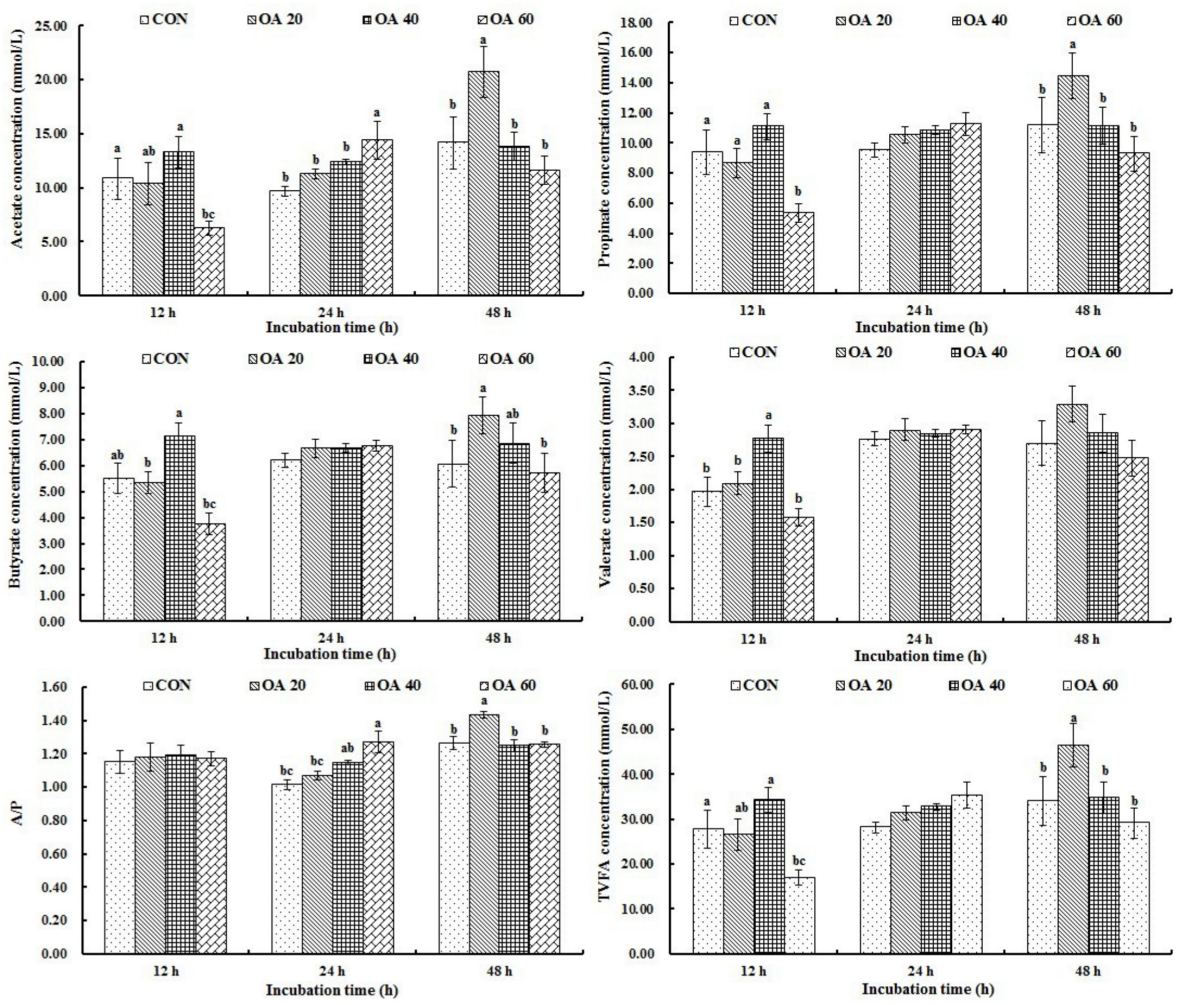
Effects of OA supplementation on VFAs concentrations of fermentation liquor *in vitro*. All values are mean ± SEM. ^a, b, c^ Means that are sharing different superscripts are different (*P* < 0.05) within the same incubation stage.

As shown in Fig 5, Molar concentrations of propionate, butyrate and valerate showed no differences with OA supplement, and the highest amount of acetate (*P* < 0.05) was found with 20 mg supplement at 48 h. Treatments did not affect the acetate: propionate ratio (*P* > 0.05) at 12 h time point *in vitro*, but increased (*P* < 0.05) the value in OA60 group at 24 h and in OA20 group at 48 h. Total VFA increased during this period, and higher level of VFA (*P* < 0.05) was found in OA20 group at the end of incubation.

### In vitro C18 fatty acids concentrations

The effects of OA supplementation on C18 fatty acids concentrations of fermentation liquid at different incubation time points are presented in Table 2. A downward trend in the concentrations of C18:1, c11 with respect to incubation time was observed. And an upward trend of C18:1, t11 with increasing dose of OA was found from the culture contents at 12 h. C18:0 of CON and OA60 reached its maximum level at 12 h and then declined all the time, respectively. C18:1, c12 of OA20 showed a negative correlation with respect to incubation time, and the maximum value at 12 h was higher (*P* < 0.05) than the minimum value at the end of incubation stage. C18:1,c13, total cis-MUFA, total PUFA, total CLA and total trans of OA60 reached their the maximum value and were higher (*P* < 0.05) than CON at 12 h, and which showed a negative correlation with respect to incubation time. The concentration of C18:2, t11t13 in all the treatments was low at 12 h and 48 h, and not detected in fluid at 24 h.

**Table 2:** C18 fatty acids concentrations at different fermentation times (g/100 mL fermentation liquid).

Items	Level	Mean	Incubation time (h)			SEM^1^	Significance effect	
			12	24	48		linear	Quadratic
C18:0	0	0.70	0.99	0.61	0.50	0.245	0.27	0.33
	20	0.64	0.59	0.77	0.56			
	40	0.34	0.57	0.22	0.22			
	60	0.56	0.94	0.37	0.38			
	SEM^2^	0.142						
C18:1,c11	0	0.18	0.19	0.19	0.17	0.047	0.20	0.26
	20	0.20	0.28^a^	0.17^b^	0.14^b^			
	40	0.17	0.22	0.16	0.13			
	60	0.24	0.30	0.22	0.19			
	SEM^2^	0.02						
C18:1, t11	0	0.03	0.03^β^	0.03	0.02	0.030	0.08	0.93
	20	0.05	0.06^α^^β^	0.05	0.04			
	40	0.05	0.06^α^^β^	0.04	0.06			
	60	0.07	0.11^α^	0.05	0.05			
	SEM^2^	0.017						
C18:1,c12	0	0.03	0.03	0.04	0.02	0.009	0.05	0.95
	20	0.03	0.05^a^	0.04^a^^b^	0.01^b^			
	40	0.04	0.03	0.05	0.04			
	60	0.04	0.04	0.05	0.04			
	SEM^2^	0.005						
C18:1,c13	0	0.11	0.08^β^	0.21	0.03	0.104	0.29	0.07
	20	0.03	0.05^α^^β^	0.03	0.02			
	40	0.03	0.03^α^^β^	0.04	0.03			
	60	0.22	0.57^α^^a^	0.04^b^	0.04^b^			
	SEM^2^	0.069						
C18:2,c9c12	0	0.43	0.48	0.58	0.23	0.329	0.60	0.10
	20	0.19	0.26	0.18	0.13			
	40	0.17	0.22	0.14	0.15			
	60	0.59	1.33^a^	0.28^b^	0.16^b^			
	SEM^2^	0.190						
C18:2,t11t13	0	0.03	0.03	-	0.02	0.003	0.01	0.05
	20	0.02	0.02	-	0.02			
	40	0.02	0.02	-	0.02			
	60	0.02	0.02	-	0.02			
	SEM^2^	0.002						
TSFA	0	4.63	6.03	3.45	4.39	1.257	0.47	0.41
	20	4.64	4.83	4.62	4.48			
	40	3.11	3.75	2.10	3.47			
	60	4.34	6.02	3.58	3.43			
	SEM^2^	0.726						
Tcis-MUFA	0	1.32	1.40^β^	1.50	1.05	1.098	0.02	0.54
	20	1.92	1.97^α^^β^	2.61	1.18			
	40	2.17	1.92^β^	3.53	1.07			
	60	3.56	5.13^α^^a^	3.93^a^^b^	1.62^b^			
	SEM^2^	0.634						
TPUFA	0	0.58	0.61^β^	0.74	0.40	0.599	0.43	0.14
	20	0.29	0.36^β^	0.27	0.24			
	40	0.24	0.29^β^	0.22	0.21			
	60	1.01	2.37^α^^a^	0.39^b^	0.27^b^			
	SEM^2^	0.346						
TCLA	0	0.04	0.04^β^	0.07	0.03	0.181	0.11	0.16
	20	0.02	0.02^β^	0.02	0.02			
	40	0.02	0.03^β^	0.03	0.02			
	60	0.28	0.79^α^^a^	0.03^b^	0.02^b^			
	SEM^2^	0.104						
Tn-3PUFA	0	0.09	0.09	0.10	0.09	0.025	0.75	0.05
	20	0.06	0.07	0.06	0.05			
	40	0.06	0.06	0.06	0.05			
	60	0.09	0.12	0.09	0.05			
	SEM^2^	0.016						
Tn-6PUFA	0	0.45	0.48^α^^β^	0.58	0.28	0.434	0.47	0.12
	20	0.21	0.27^β^	0.18	0.18			
	40	0.17	0.22^β^	0.14	0.16			
	60	0.74	1.73^α^^a^	0.28^b^	0.20^b^			
	SEM^2^	0.251						
Ttrans	0	0.13	0.12^β^	0.13	0.13	0.234	0.09	0.44
	20	0.21	0.22^β^	0.27	0.16			
	40	0.18	0.13^β^	0.20	0.23			
	60	0.49	0.93^α^^a^	0.32^a^^b^	0.22^b^			
	SEM^2^	0.146						

^α, β^ Within a column, means with different Greek superscripts are different (*P* < 0.05);

^a, b^ within a row, means with different superscripts are different (*P* < 0.05).

SEM^1^ for treatment × incubation time; SEM^2^ for pooled standard error of the means over levels.

## Discussion

*Leymus chinensis* has been widely used as forage for ruminant animals in China, but there is rarely as a fermentation substrate *in vitro*. In current study, the reduction of *Leymus chinensis* IVDMD after the treatment of OA addition was in agreement with previous studies [3]. In addition, there are studies also found that dietary supplementation with vegetable oils or fish oil rich in unsaturated fatty acids resulted in a reduction of DMI in ruminants [23–24]. Unsaturated fatty acids have been reported to have a negative effect on fiber digestibility due to an antimicrobial effect in the diet on rumen function *in vivo* and *in vitro* [25]. Additionally, Jalc *et al* [3]. reported that adding of OA up to 3.5% in DM to a lucerne-barley (80:20) diet, although not reaching a significant level could reduce *in vitro* degradability of dry matter. The lower intake or lower fiber digestibility on the diet might be related to negative effects on rumen function due to the effects of lipid on rumen bacteria.

Previously, Lee *et al* [26]. studied the effect of long chain fatty acids on *in vitro* gas production by rumen anaerobic fungus and confirmed that *in vitro* gas production of filter paper was increased by the addition of oleic acid (OA) with an increase of gas production rate. However, Zhang *et al* [6]. found that *in vitro* gas production of mixed wild rye meal and corn meal at 24 h using mixed rumen fluids of sheep was decreased by the addition of different levels of OA. In this study, the addition of OA decreased the *in vitro* gas production of *Leymus chinensis* with a decrease of vf and FRD_0_ and an increase of t_0.5_. This would be ascribed to that dietary UFA supplementation could suppress the density and activity of rumen bacteria, particularly the cellulose-utilizing bacteria [27]. Previous studies on GP have given some controversial results which depend on different substrates, dosages of supplemented long chain fatty acids and associated rumen microbes. According to our study the OA decreased GP with the substrate of *Leymus chinensis*, and that higher dose of OA tended to decrease more GP.

Studies have been focused on how to reduce the rumen methanogenesis [28], since methane production from the enteric fermentation (natural digestive process in the rumen) can contribute to the energy loss and greenhouse gases emissions. Ding *et al* [29]. found that adding coconut oil to the diets at a level of 12 g/day reduced CH_4_ production by 61.3% compared with the control in Tibetan sheep. Moreover, O’Brien *et al*.[30] reported that addition of OA in two feed types (ryegrass or grass silage supplemented with barley grain) reduced methane production *in vitro*, separately. Similarly, our present study observed that addition of OA to *Leymus chinensis* reduced methane production, and the reduced amount of methane differed depending on the adding level of OA. This inhibition may be due to a toxic effect of OA towards methanogenic archaea.

Feeds are normally fermented in the rumen to produce H_2_ and volatile fatty acids. Methanogens use H_2_ and carbon dioxide to form CH_4_ which will be released from the rumen into the atmosphere [31]. The H_2_ concentration can regulate the rate of CH_4_ production in rumen. Published studies showed that partial inhibition of methanogens results in higher H_2_ concentrations, and less CH_4_ formation [32]. At the present study, addition of OA reduced CH_4_ production, but increased H_2_ production, this was consistent with the previous study [6]. The reason may be that CH_4_ production can be inhibited by addition of unsaturated fatty acid due to a direct toxic effect on rumen methanogens.

Jalc *et al*.[33] found that all supplemented diets with plant oils rich in unsaturated fatty acids increased pH compared to the control *in vitro*. Whereas, Jalc *et al*.[5] used OA as supplements (3.5% wt/wt) to a diet containing 80% lucerne and 20% barley and found that pH value was decreased by OA supplement compared with control *in vitro*. In the current study, we found that pH value of fermentation liquid was also different at different fermentation time points. This may be due to large amount of hydrogen ions offered by OA or some other generated acids reduced pH at the first stage, and then, OA addition inhibited microbial degradation of the substrate and increased pH at 24 h, eventually the accumulation of VFA might lead to the decline of pH at 48 h under the conditions of *in vitro* fermentation. Addition of OA inhibited rumen microbes, and limited the deamination of proteins and peptides, finally led to a significant decrease of NH_3_-N concentration compared with the control *in vitro* at 12 h. Our present data showed no significant difference in the NH_3_-N concentrations among treatments *in vitro* at 24 and 48 h, which suggest that the biohydrogenation mainly occurred during the first 12 h *in vitro*.

Pilajun and Wanapat [34] used the diets supplemented with coconut oil at 50 g/kg DM to feed swamp buffalo bulls and found that acetic acid and TVFA were decreased while propionic acid was increased. A past study found that the acetate to propionate ratio was increased significantly, and TVFA was decreased by OA supplementation [5]. However, Hristov *et al*. [35] reported that coconut oil supplementation did not affect the proportion of propionic acid in the rumen of the lactating cows. In addition, Wanapat *et al*. [36] supplemented vegetable oils (coconut oil and sunflower oil) in a ratio of 50:50 at 6% of concentrate during a 5-month feeding trial and found decreased proportion of propionic acid, but increased the acetic acid to propionic acid ratio in the rumen. These variations could be caused by different fatty acids, adding amounts in the diets and experimental animals. In present study, we're not accounting for the production of isobutyric acid and isovaleric acid so that the gas production and the total VFA production. Finally, we got the trends the production of VFAs were different at different time point. These may be because of the microbes, the production of VFAs and the dose of OA changed with the incubation *in vitro*.

The fate of dietary UFA in the rumen is associated with biohydrogenation process. The OA, a typical fatty acid often used in ruminant diets, is usually described as being hydrogenated directly to stearic acid without the formation of intermediates. However, OA is not completely saturated to stearic acid, but isomerized to other acids of the C18:1 family [37]. Previous work by Jenkins *et al*. [38] also reported that OA is the ruminal precursor of hydroxystearic acid (HSA) and the HSA could be converted to other fatty acids in the rumen. We observed that addition of OA resulted in the increases of some acids of C18:1 family and trans compared with the control. Conjugated linoleic acids (CLA) are found mostly in the meat and dairy products derived from ruminants that possess a range of beneficial health effects in animal models [39]. Hristov *et al*. [40] investigated the effects of replacing conventional, solvent-extracted canola meal with high-oleic acid canola on milk fatty acid composition in lactating dairy cows, and found total monounsaturated fatty acid (TMUFA), total polyunsaturated fatty acids (TPUFA) and CLA were increased significantly compared with the control. Similarly, The levels of the main CLA isomers (cis9, trans11 C18:2, represented a 50–70% proportion of the total CLA) significantly increased (32.8%) after OA as supplements (3.5% wt/wt) to a diet containing 80% lucerne and 20% barley *in vitro* [5]. In our present study, the C18:2, t11t13 was not detected in rumen fluid, and the reasons may be that it was biohydrogenated by microbes and converted to other substances. Overall, these findings are the similar with our study, and indicate that the amount of OA supplement in the diet, as well as the incubation time *in vitro*, have a marked effect on the changes in rumen fluid total CLA concentrations. The inconsistency in fermentation parameters sourced from different studies would be ascribed to different types of FA (different plant oils), dose of supplement, sampling time, experimental animal and substrates, etc.

## Conclusion

Supplementing different doses of OA to *Leymus chinensis* decreased the GP and suppressed CH_4_ emissions *in vitro*. Furthermore, there is a trend of increments of the beneficial fatty acids by OA supplementation. If the mechanisms of ruminal lipid metabolism to OA in the diet can be identified and controlled, it could be possible to develop nutritional strategies for ruminants with a properly balance between negative effect (*e*.*g*. reduction in IVDMD) and positive effects (*e*.*g*. reduction in CH_4_ and productions with high levels of trans-11 18:1 and CLA).
